# Nurse prescribing practices across the globe for medication-assisted treatment of the opioid use disorder (MOUD): a scoping review

**DOI:** 10.1186/s12954-023-00812-y

**Published:** 2023-06-23

**Authors:** Sonam Prakashini Banka-Cullen, Catherine Comiskey, Peter Kelly, Mary Beth Zeni, Ana Gutierrez, Usha Menon

**Affiliations:** 1 School of Nursing and Midwifery, Trinity College Dublin, Dublin, Ireland; 2 Florida Department of Health, Tallahassee, USA; 3 Health College of Nursing, University of South Florida, Tampa, USA

**Keywords:** MOUD, Medication-assisted treatment, Opioid use disorder, Nurse prescriber, Scoping review

## Abstract

**Background:**

Despite the dramatic increase in opioid-related deaths in recent years, global access to treatment remains poor. A major barrier to people accessing Medication-assisted treatment of the opioid use disorder (MOUD) is the lack of providers who can prescribe and monitor MOUD. According to the World Drug Report, more young people are using drugs compared with previous generations and people in need of treatment cannot get it, women most of all. Nurse prescribers have the potential to enhance both access and treatment outcomes. Nurse prescribing practices do, however, vary greatly internationally. The aim of this scoping review is to explore nurse prescribing practices for MOUD globally with a view to informing equitable access and policies for people seeking MOUD.

**Methods:**

This scoping review was informed by the preferred reporting items for systematic reviews and meta-analysis extension for scoping reviews (PRISMA-ScR). Electronic searches from 2010 to date were conducted on the following databases: PsycInfo, PubMed, Embase, and CINAHL. Only studies that met the eligibility criteria and described nurse prescribing policies and/or behaviours for MOUD were included.

**Results:**

A total of 22 articles were included in the review which found several barriers and enablers to nurse prescribing of MOUD. Barriers included legislation constraints, lack of professional education and training and the presence of stigmatizing attitudes. Enablers included the presence of existing supportive services, prosocial messaging, and nurse prescriber autonomy.

**Conclusion:**

The safety and efficacy of nurse prescribing of MOUD is well established, and its expansion can provide a range of advantages to people who are dependent on opiates. This includes increasing access to treatment. Nurse prescribing of MOUD can increase the numbers of people in treatment from ‘hard to reach’ cohorts such as rural settings, or those with less financial means. It holds significant potential to reduce a wide range of harms and costs associated with high-risk opiate use. To reduce drug-related death and the global burden of harm to individuals, families, and communities, there is an urgent need to address the two key priorities of nurse prescriber legislation and education. Both of which are possible given political and educational commitment.

## Background

Worldwide, drug use has led to about 500,000 deaths in 2019, with over 70% of these being related to opioids, and more than 30% of those deaths caused by overdose [1]. In 2020, the USA saw over 91,000 drug overdose deaths, of which 75% were from opioids [2]. Though opioid-related deaths have increased significantly, access to treatment remains suboptimal. Medication-assisted treatment for opioid use disorder (MOUD) has been shown to effectively treat dependence on opioids; however, there are a limited number of providers who can prescribe and monitor MOUD. In most countries, nurses exceed the number of physicians, and many provide care in areas that are medically underserved.

Nurse prescribers for MOUD have the potential to enhance access to treatment and treatment outcomes. However, nurse prescribing practices vary greatly internationally as do levels of education and certification. In the North American region (USA and Canada), the highest level of advanced practice for nurses is certified nurse practitioners. The countries that currently have well to moderately developed nurse practitioner roles included in workforce planning are Australia, Canada, Finland, Ireland, the Netherlands, New Zealand, the United Kingdom (England, Northern Ireland, Scotland, and Wales), and the USA [3].

In other countries, there may be varying levels of practice, but many are informal roles based on need and experience. Standardization of the nurse role in MOUD may be important to achieve the goal of reducing preventable deaths. However, as a critical first step, the aim of this scoping review is to explore nurse prescribing practices for MOUD globally. Findings will inform the need to develop and implement policy and to integrate guidelines for practice internationally.

## Methods

### Scoping review

A scoping review, as defined by Arksey and O’Malley [4], is a method of mapping “rapidly the key concepts underpinning a research area and the main sources and types of evidence available. Such reviews can be undertaken as standalone projects in their own right, especially where an area is complex or has not been reviewed comprehensively before” [5]. This review will seek to rapidly uncover the main sources and types of evidence available in the area of nurse prescribing practices for MOUD. In addition, the framework developed expanded on this definition of a scoping review by identifying four main reasons for conducting a scoping study: (1) to examine the extent, range, and nature of research activity; (2) to determine the value of undertaking a full systematic review; (3) to summarize and disseminate research findings; and (4) to identify research gaps in the existing literature. It has been highlighted that scoping studies differ from systematic reviews because authors do not typically assess the quality of included studies in scoping reviews [4]. Scoping studies also differ from narrative or literature reviews in that the scoping process can require analytical reinterpretation of the literature [6]. Grant and Booth [7] in their typology of reviews describe scoping reviews as providing a preliminary assessment of the potential size and scope of available research in the literature, with the aim of identifying the nature and extent of research evidence including ongoing research. Within this review, the focus will be on reasons (1) and (4) above. To ensure rigor and credibility in the process, the review was conducted according to the preferred reporting items for systematic reviews and meta-analyses extension for scoping reviews (PRISMA-ScR) checklist.

### Search strategy, eligibility criteria, extraction, and synthesis

This review used a population, exposure, outcomes (PEOS) framework to assess the eligibility of the studies (see Table 1 below) [8]. National and international studies published between 2010 and 2021 were included, and the review was limited to English language articles. The search strategy was piloted to check the appropriateness of keywords and databases. The following databases were searched: PsycInfo, PubMed, Embase, and CINAHL. All articles were uploaded into Endnote Version X9 software, and duplicates were identified and removed. The articles were then uploaded into COVIDENCE for screening. The first phase of screening was conducted by two researchers based on title and abstract, and the second phase of screening was conducted by all researchers based on full text.

**Table 1:** PEOS Framework

Population	Nurse prescribers
Exposure	Opioid use
Outcomes	Prescribing practices
Study	Quantitative and qualitative research design

The studies that met the inclusion criteria below were included in the review:

- Studies on nurse prescribing practices.
- Studies on MOUD.
- Studies in English language.
- Studies between 2010 and 2021.

The studies that did not meet the inclusion criteria were excluded from the review. The study was not restricted to either qualitative or to quantitative studies; however, opinion articles and reviews were excluded. The data extraction was conducted using EndNote Version X9 [9] and COVIDENCE [10]. Data extracted from the eligible studies were then synthesized narratively. A grey literature search was not conducted as part of this review.

## Results

**Fig. 1:**
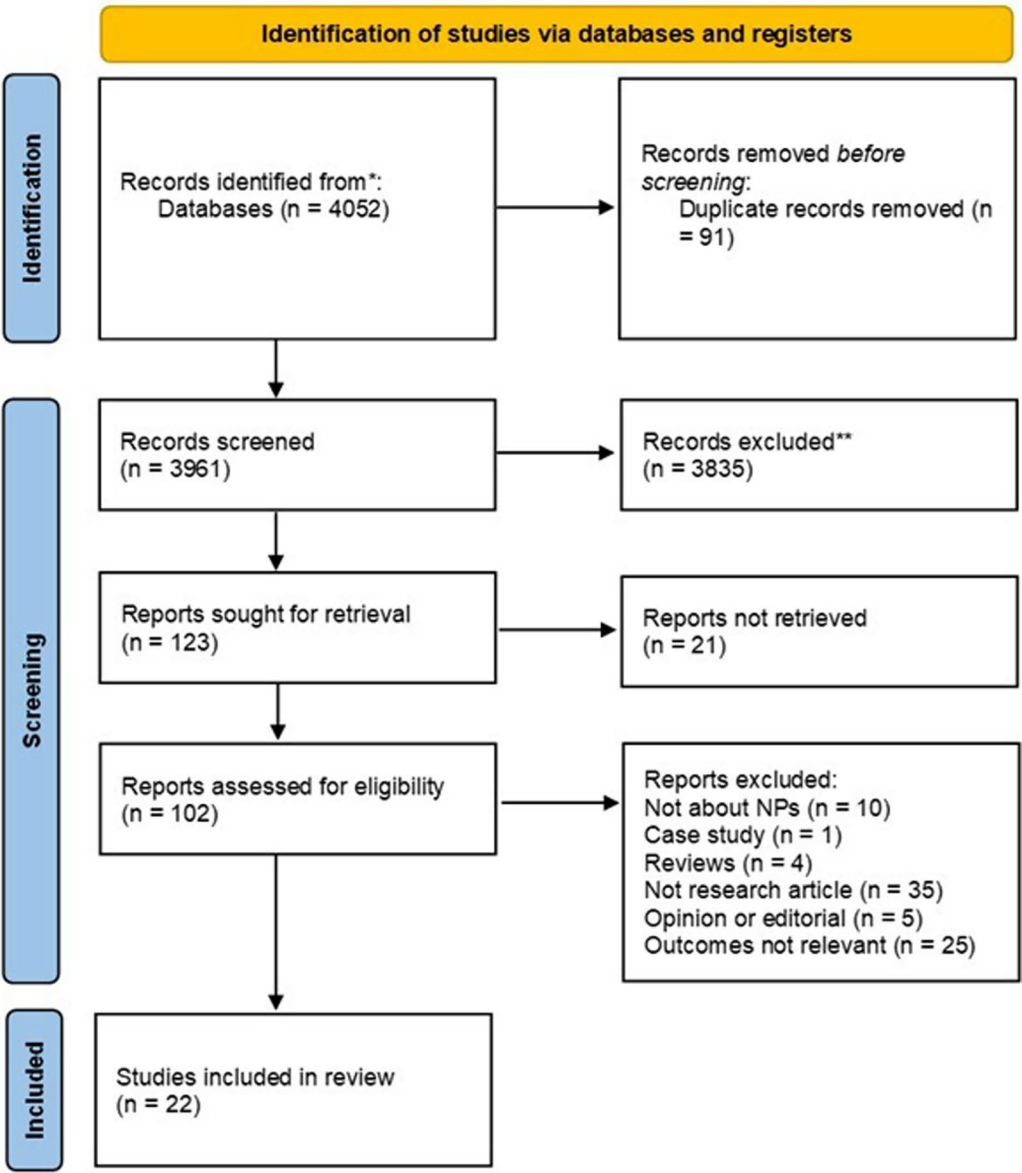
PRISMA search flow

A total of 4052 articles were retrieved from the database searches of which 91 duplicates were removed, resulting in 3961 articles for the title and abstract screening phase. Following the first round of screening based on title and abstract, a total of 3835 articles were removed and a further 21 were excluded as full texts could not be retrieved. The second round of screening of the 102 full texts was conducted by four researchers, and 22 articles were deemed eligible for inclusion. Figure 1 shows the PRISMA search flow, and Table 2 provides a summary of the articles included in the scoping review.

**Table 2:** Eligible studies and its characteristics

Author	Title	Year	Country	Study design
Andrilla et al.	Prescribing practices of nurse practitioners and physician assistants waivered to prescribe buprenorphine and the barriers they experience prescribing buprenorphine	2020	USA	Survey design
Andrilla et al.	Geographic distribution of providers with a DEA Waiver to prescribe buprenorphine for the treatment of opioid use disorder: a 5‐year update	2019	USA	Secondary data analysis
Andrilla et al.	Projected contributions of nurse practitioners and physician’s assistant to buprenorphine treatment services for opioid use disorder in rural areas	2020	USA	Secondary data analysis
Auty et al.	Buprenorphine waiver uptake among nurse practitioners and physician assistants: the role of existing waivered prescriber supply	2020	USA	Secondary data analysis and survey design
Barnett et al.	In Rural areas, Buprenorphine Waiver adoption since 2017 driven by nurse practitioners and physician assistants	2019	USA	Secondary data analysis
Bateman et al.	Psychopharmacological treatment of young people with substance dependence: a survey of prescribing practices in England	2014	England	Survey design
Bates et al.	Facilitators and barriers to nurse practitioners prescribing methadone for opioid use disorder in nova scotia: a qualitative study	2021	Canada	Qualitative study design
Carroll	Implementation of office-based buprenorphine treatment for opioid use disorder	2021	USA	One-group post-test-only design
Comiskey et al.	Clients’ views on the importance of a nurse-led approach and nurse prescribing in the development of the healthy addiction treatment recovery model	2019	Ireland	A cross-sectional survey
Dieujuste et al.	Provider perceptions of opioid safety measures in VHA emergency departments and urgent care centers	2021	USA	Survey design
Domino et al.	Nudging primary care providers to expand the opioid use disorder workforce	2021	USA	Experimental design
Elliott et al.	Changing nurse practitioner students' attitudes and beliefs about caring for those with opioid use disorders	2021	USA	Quasi-experimental design
Jones et al.	Empowering psychiatric mental health nurse practitioners to expand treatment opportunities for veterans with opioid use disorder	2020	USA	Survey design
Kameg et al.	Technology based educational approaches to address opioid use management by advanced practice registered nurses	2020	USA	Mixed methods survey design
Klein et al.	The geographic impact of buprenorphine expansion to nurse practitioner prescribers in Oregon	2020	USA	Secondary data analysis
Nguyen et al.	The association between scope of practice regulations and nurse practitioner prescribing of buprenorphine after the 2016 opioid bill	2021	USA	Secondary data analysis
Roehler et al.	Buprenorphine prescription dispensing rates and characteristics following federal changes in prescribing policy, 2017–2018: a cross-sectional study	2020	USA	Secondary data analysis
Saunders et al.	Medicaid participation among practitioners authorized to prescribe buprenorphine	2021	USA	Secondary Data Analysis
Sorrell et al.	From policy to practice: pilot program increases access to medication for opioid use disorder in rural Colorado	2020	USA	Non-randomized intervention
Spetz et al.	Barriers and facilitators of advanced practice registered nurse participation in medication treatment for opioid use disorder: a mixed methods study	2021	USA	Mixed methods
Spetz et al.	Nurse practitioner and physician assistant waivers to prescribe buprenorphine and state scope of practice restrictions	2019	USA	Secondary data analysis
Wakeman et al.	A hospital-wide initiative to redesign substance use disorder care: Impact on pharmacotherapy initiation	2021	USA	Secondary data analysis

A narrative synthesis of the 22 eligible studies was conducted, and six key topics were identified. Table 3 provides an overview of these key topics and summary statements. Within this, *waiver uptake* relates to studies conducted in the USA, where practising nurse prescribers, who may not be specialists in the addictions field, must apply for a legal waiver to prescribe MOUD.

**Table 3:** Key topics and summary statements

*Role of NPs and prescribing practices*
Nurse prescribing is at various stages of development across jurisdictions in terms of; training, prescribing autonomy and scope of practice
*The importance of prescribing legislation*
There is a direct relationship with passing legislation to allow nurse prescribing of MOUD and increasing access to, and increasing numbers in MOUD treatment
*Underserved regions: access, support and needs*
Where there are more nurse prescribers, people living in rural areas and those receiving state-supported care are more likely to access MOUD treatment
*Importance of practice and institutional support*
Access to institutional support and education for nurse prescribers will increase the numbers of prescribing nurses
*External factors influencing NP uptake*
There is higher NP uptake in areas where there is; pro-social messaging from recruiters, already high numbers of NPs, and greater practitioner autonomy [i.e., no medical oversight]
*Barriers to uptake of MOUD; NP’s perceptions of people who are dependent on opiates*
Negative attitudes of prescribers towards people who are dependent on opiates and social stigma. Perceived complexity, e.g., chronic health issues, risk of violence, drug use and diversion of prescribed medication

### Role of NPs and prescribing practices

The role of nurse practitioners and their prescribing practices varies across countries. Broadly, the term nurse practitioner (NP) refers to a type of advanced practice registered nurse (APRN) and is used interchangeably with the title of advanced nurse practitioner (ANP) and advanced registered nurse practitioner (ARNP), although the preferred legal term varies by area. To become an APRN, individuals must obtain either a Master of Science in Nursing (MSN) or a Doctor of Nursing Practice (DNP). In the USA, the 2016 Comprehensive Addiction and Recovery Act (CARA) allowed NPs to begin prescribing MOUD [11]. The legislation also allows Physicians’ Assistants (PAs) to prescribe MOUD. While PAs undergo different training and licensing and are not nurses, they often work alongside NPs. The role of NPs varies further with some states in the USA, allowing NPs to practice autonomously and others requiring physician oversight. Similarly, Canada grants NPs prescribing privileges for MOUD at the federal level, but specific requirements vary by province [12]. In England, where the title ‘nurse practitioner’ is not legally protected, trained nurses may also prescribe MOUD autonomously [13]. Other countries, such as Ireland, are at an earlier stage in expanding the role of nurse practitioners in MOUD. Research by Comiskey and colleagues [14] highlighted that there is a clear need to expand the role of NPs into addiction services from a nursing policy development perspective. They also reported that clients from the addiction services strongly expressed the need for nurses’ role to be expanded in terms of treatment plan, specifically methadone treatment pathways, procedures, prescription, and dosage [14].

### The importance of prescribing legislation

New MOUD NPs in the USA, i.e., NPs who are legally allowed to prescribe opioids to treat OUD, are prescribing to more patients than doctors and therefore, providing much-needed treatment [15], especially for those in rural areas. In another study by Andrilla and colleagues [16], it was reported that the estimated number of treated patients by NPs and PAs per 10,000 population increased from 15.4 to 17.7. This highlights that NPs newly eligible to prescribe have the potential to reduce access problems that many patients are currently facing, quite significantly [17]. Klein and colleagues [18] also found that the impact of adding nurses as authorized prescribers has enhanced access to treatment, more specifically buprenorphine prescriptions, in very rural and sparsely populated areas where patients were severely underserved for MOUD. Both studies by Roehler [19] and Saunders [20] highlight that NPs are especially important in filling treatment gaps for patients in underserved areas and populations. Another study reported that patients seen by nurses in office-based addiction treatment in the primary care setting were more likely to be stable than patients in the hospital or transitional clinic, which emphasizes the importance of nurses providing addiction treatment in primary care [21].

### Underserved regions: access, support and needs

According to research by Andrilla and colleagues [16], NPs are a significant part of the rural primary care workforce and allowing NPs to prescribe buprenorphine for OUD will expand access to treatment access. However, there are many barriers that affect NP waiver uptake. The study also highlighted the need to provide practice support and solutions to barriers which have previously been identified by physicians [16]. Andrilla and colleagues suggested that despite the increase in numbers of MOUD prescribers between 2012 and 2017, many rural areas lacked MOUD providers [22]. As a result, rural communities experience greater barriers to receiving care and higher rates of overdose than urban areas [22]. Roehler and colleagues found an increase in NPs and PAs was 1.3 times higher in rural areas compared to urban areas, which indicates the importance of NPs and PAs in expanding access to buprenorphine in rural areas [19]. While Barnett and colleagues reported that in rural areas, broad scope-of-practice regulations were associated with twice as many registered NPs per 100,000 population compared to restricted scopes of practice [23]. State-funded programs in the US have been vital in addressing the opioid epidemic, research by Saunders and colleagues found that NPs are twice more likely to prescribe buprenorphine to state-funded patients and thrice more likely to prescribe to a large number of state-funded patients in comparison with physicians [20]. The study also suggests that as a result, NPs are especially important in filling treatment gaps in underserved regions [20].

### Importance of practice and institutional support

A recent study highlighted that there is a need for ongoing learning resources and support for NP practice [12]. Participants in the study had varying opinions regarding the availability and accessibility of methadone supports for NPs, while some felt that there is a lack of institutional support, others were unsure about access to methadone education [12]. Research by Sorrel and colleagues evaluated the effectiveness of a pilot program at the University of Colorado College of Nursing, which provided a community network of practice and monthly virtual conferencing sessions to discuss organizational best practices and challenges in delivering MOUD [24].

NPs identified lack of adequate expertise in working with MOUD patients as a barrier. The structure of NP education was also found to be discouraging as NPs felt that they received little addiction training and no methadone education as part of their initial training. Addiction studies are also seen as a niche area requiring specialized and difficult knowledge [12], and this can deter prescribers from the role. As highlighted previously, NPs also reported a lack of institutional and practice support [12, 25], while other research highlighted that the lack of support services such as mental health and other psychosocial support services for patients with OUD posed as a key barrier [15]. This relates back to lack of support and specialty backup for working with OUD patients with complex needs. It was also found that educating NPs on MOUD and empowering them has the potential to increase access to a sample of veterans, which suggests that education can act as a key enabler [26]. NPs have reported feeling a lack of confidence in their ability to manage OUD [15], however, as highlighted by Elliot and colleagues, educational experiences and exposure to working with OUD patients can help NPs develop more confidence [27].

### External factors influencing NP uptake

The study by Carroll evaluated the implementation of NP led MOUD clinics and found that the pilot programme increased MOUD access by 34% (*n* = 21) over 7 months [28]. The study included participants who attended at least one appointment at the nurse-led weekly Buprenorphine-Medically Assisted Treatment (B-MAT) clinic using a low-threshold, chronic-care model for the treatment of OUD [28]. The outcomes suggest that NP-led MOUD clinics are feasible and safe, and ANPs are well positioned to provide MOUD; however, only 4% of NPs have applied for the waiver to prescribe for MOUD since 2016. In this section, some of the key enablers and barriers to NPs uptake is further discussed.

#### Existing prescribers and MOUD services

Recent study reported that the uptake among NPs is strongly associated with existing prescriber supply. An increase in supply was observed most substantially in states with an already-high supply. It was also found that NP uptake was significantly greater in states that had more Opioid Treatment Programs (OTP) per capita. Non-OTP facilities offering any MOUD were also significantly associated with higher uptake [17].

#### Recruitment strategies

Methods of recruiting and promoting uptake can have a significant influence on NPs. Domino and colleagues conducted a study to evaluate the use of three methods to recruit NPs in the OUD workforce [29]. Three types of recruitment messaging were used: (a) prosocial messaging, (b) compensation messaging, and (c) prosocial and compensation messaging. They found that NPs responded the most to prosocial and compensation messaging and represented 61% increase over the physician response rate, followed by prosocial messaging only [29].

#### Autonomy, restrictive scope of practice and resistance from practice partners

Rural NPs reported facing more resistance from their practice partners in comparison with urban NPs [15]. This was also reported by Bates and colleagues [12], while Barnett and colleagues found that regions with full NP scope of practice regulations were associated with twice the number of NPs [23]. Having full NP scope of practice regulations means that NPs do not require physician oversight for prescribing, diagnosing, or treating patients. A recent study by Kameg reported that the primary barrier to prescribing buprenorphine was scope of practice limitations, as reported by 21.5% of respondents [25]. It was also reported that ANPs often refer to bureaucratic issues such as procuring collaborating or supervising physicians [25]. While the 2016 CARA has been a step forward for expanding MOUD access by allowing NPs to prescribe for OUD, however, Nguyen and colleagues found that the impact of CARA has been limited. The study found that NPs account for relatively small proportions of buprenorphine prescriptions; however, a greater percentage of patients received MOUD from NPs in states that have a less-restrictive scope of practice [30]. This suggests that physician oversight slowed the provision of MOUD and the growth of NPs [30]. Another recent study found that higher number of ANPs were registered in states where they could prescribe without physician oversight [31]. Consequently, a key influencing factor for NP uptake is autonomy in practice and this was found to be strongly associated with NP uptake [17, 31, 32].

### Barriers to uptake of MOUD: NP’s perceptions of people who are dependent on opiates

This section highlights the negative attitudes of prescribers towards people who are dependent on opiates and social stigma. It also highlights the perceived complexity of MOUD prescribing, e.g., chronic health issues, risk of violence, drug use and diversion of prescribed medication.

#### Prescriber and public stigma

A study in Canada found that personal beliefs of NPs often pose a barrier to providing treatment, for example, views such as patients with OUD deserve less care than other patients [12], this was also reported as a barrier in the study by Spetz and colleagues [31]. Participants on the study acknowledged that some prescribers would avoid prescribing methadone for personal reasons [12]. NPs also discussed that public stigma remains a significant barrier, one stating that “there’s stigma of just going every day to the pharmacy and being there, exposed, people staring at you…” Another NP reflected that stigma may be addressed by educating prescribers and increasing their experience of working with people with OUD. It was also reported that patient’s willingness and lack of education regarding MOUD presented as a barrier to practice [33]. Elliot and colleagues [27] conducted a quasi-experimental study in which five NP doctoral students attended lectures and 16 h of direct clinical experience with OUD patients. Students reported positive attitude changes and personal reflections which suggest that such educational experiences can be beneficial for developing more confident, skilled and compassionate NPs to address the opioid crisis.

#### Complexity of patients living with OUD

Andrilla and colleagues reported that NPs identified lack of specialty backup for complex patients as a key barrier to their practice [15]. Bates and colleagues found that providers are reluctant to assume the care of OUD patients with multiple chronic health challenges [12]. NPs also highlighted that little incentives exist to begin working with new, complex OUD patients, and perceived complexity of patient needs may deter NPs from prescribing methadone [12]. As well as complex client needs, NPs reflected on the risk of violence due to MOUD as a barrier to their practice [12]. Diversion and drug use was also found to be a key barrier for NPs [15].

## Discussion and conclusion

The safety and efficacy of nurse prescribing of MOUD is well established, and its expansion can provide a range of advantages to people who are dependent on opiates. This includes increasing access to treatment, but nurse prescribing of MOUD can increase the numbers of people in treatment from ‘hard-to-reach’ cohorts such as those in rural settings, or those with less financial means [19, 34]. This in itself holds a significant potential to reduce a wide range of harms and costs associated with high-risk opiate use [35, 36]. Developing NP of MOUD can also help to create new and innovative treatments which can allow services such as detoxification for complex clients, normally only considered appropriate for in-patient settings, to be delivered in a person’s own home [37]. Where MOUD treatment is already available, it is likely that developing NP will also provide opportunities for enhanced key working and more responsive services [38]. Within England and Scotland, it has been found that the number of non-medical prescribers has grown considerably in the recent past and this has provided an opportunity for nurses particularly in England to work at an advanced level [39].

The studies included in this review, although mostly from the USA, are reflective of the European context, in that the development of nurse prescribing of MOUD is subject to the efforts made within each jurisdiction to progress it. There are significant variations across regions in terms of levels of training, autonomy and scope of practice and indeed whether nurse prescribing of MOUD happens *at all* [40]. For example, in the UK, nurses can prescribe MOUD independently, but ‘nurse practitioner’ is not a legally protected title as it is in other regions [41]. In this respect, the already established potential and recognition of the role of NP of MOUD has yet to be realized globally. Recent initiatives such as the ‘safer supply’ policy in British Columbia in Canada provide good examples of how the nursing workforce can provide service users access to range of MOUD treatments including injectable medications [42]. Given the increasing international policy focus placed on expanding access to harm reduction interventions such as methadone, which reduce drug-related deaths [43], it is imperative that initiatives such as NP of MOUD be fully recognised and developed by legislators, policymakers and planners. In this context, there is some guidance available that clarifies the NP role and illustrates the advantages of NP to non-experts [44]. Developing greater international consensus on this, bolstered by more research, and better ‘marketing’ of the NP model would enhance awareness of the advantages of NP of MOUD even further [40].

To build on current success, the expansion of NP of MOUD also requires ‘whole-systems’ support. In the first instance, this should start with passing the necessary legislation to allow nurse prescribing to take place [16]. Secondly, in order to ensure maximum uptake and to optimise positive impacts on service users, this legislation should allow NPs to prescribe autonomously [12, 23]. Both third-level institutions and healthcare providers also need to collaborate on how to provide the most appropriate institutional training and support, and this should incorporate ongoing education and ‘in-practice’ supervision [45, 46]. Where relevant, this education and supervision should aim to address negative attitudes of non-specialist prescribing nurses towards people who use drugs [47]. More broadly, this should involve delivering addiction education and ‘pro-social’ messaging into the nursing ‘water supply’ at the undergraduate and postgraduate levels [48]. Assurances should also be provided to potential practitioners by properly resourcing ‘joined-up’ services with adequate clinical governance and appropriate input from multi-disciplinary teams which can support practitioners in caring holistically for people with complex needs [49]. These measures should, in turn, increase the uptake of non-specialist nurse prescribers to MOUD treatment and increase the desire for more nurses to specialise in this area.

### Strengths and limitations

A key strength of this review is that it focuses on the role of NPs in providing MOUD treatment. Nurse prescribing practices across the globe for MOUD vary greatly internationally as do levels of education and certification. Nurse prescribers for MOUD have the potential to enhance access to treatment and treatment outcomes, and this review highlights the enablers and barriers to NPs role in MOUD treatment. The scoping review method provides a rigorous and transparent method for mapping key research areas. To ensure rigor and credibility in the process, the scoping review was conducted according to the preferred reporting items for systematic reviews and meta-analyses extension for scoping reviews (PRISMA-ScR) checklist and the literature was cross-checked and validated by all authors. In relation to the limitations of the review, it is important to note that the majority of literature that was yielded in the review was mainly from the US which limits the generalizability of the findings. Another limitation of this study is that a grey literature search was not conducted. While scoping reviews have rigorous methods, it is a rapid form of mapping research areas and as a result, may have missed some important papers.

## Conclusion

Medication-assisted treatment for opiate use is a cost-effective and widely utilized approach which facilitates both harm reduction and recovery, but it is not always accessible to those who require it the most. Despite some inconsistencies in *how* nurse prescribing of MOUD has been developed, its effectiveness in each context is well established and there is ample evidence of appropriate strategies that can be used to support it. Nurse prescribing of MOUD can be further enhanced through the provision of targeted education and institutional support for nurse prescribers. Overall, the potential for nurse prescribing of MOUD to improve existing treatment and to provide treatment where there is none available is clear. In many jurisdictions, this potential has been realized by legislators and planners, but further realization and expansion of nurse prescribing of MOUD is required globally.

## Data Availability

The search strategy which was used to generate the data for this paper is openly available in the open science foundation repository and can be accessed at: https://osf.io/jg6xw/?view_only=8b0b1e5d117e47eb9dbfa9559f32dfb7.

## Abbreviations

MOUD: Medically assisted treatment for opiate use disorder
PEOS: Population, exposure, outcomes, study design
PRISMA-ScR: Preferred reporting items for systematic reviews and meta-analyses extension for scoping reviews
NP: Nurse practitioner
APRN: Advanced practice registered nurse
ANP: Advanced nurse practitioner
ARNP: Advanced registered nurse practitioner
MSN: Master of science in nursing
DNP: Doctor of nursing practice
CARA: Comprehensive addiction and recovery act
PA: Physicians’ assistants
OUD: Opiate use disorder
B-MAT: Buprenorphine-medically assisted treatment
